# Complex landscapes, complex diets: DNA metabarcoding reveals lady beetle prey richness increases with landcover diversity

**DOI:** 10.1002/eap.70248

**Published:** 2026-05-11

**Authors:** Benjamin Iuliano, Claudio Gratton

**Affiliations:** ^1^ Department of Natural Sciences CUNY–Baruch College New York New York USA; ^2^ Department of Integrative Biology University of Wisconsin‐Madison Madison Wisconsin USA; ^3^ Department of Entomology University of Wisconsin‐Madison Madison Wisconsin USA

**Keywords:** agroecology, biological control, diet composition, food webs, landscape ecology, molecular ecology

## Abstract

Understanding the capacity of mobile organisms such as insects to utilize resources across different patches in a landscape can reveal strategies for their conservation. Past research suggests that higher levels of non‐crop habitat or landcover diversity in agricultural regions typically benefit generalist predators who can fortify their diets with prey from multiple adjacent habitats. For some taxa such as lady beetles (Coccinellidae), dietary diversity is associated with improved fitness, but foraging patterns in real landscapes are hard to measure. We used a DNA metabarcoding approach to explore how the presence and taxonomic richness of arthropod prey in lady beetle diets varied by local habitat (crop vs. non‐crop) and landscape complexity (non‐crop habitat and landcover diversity in a 250 m radius). We collected over 500 individual lady beetles from a range of landscapes in 2019 and 2021 in southern Wisconsin (USA), performed whole‐body DNA extractions, amplified arthropod DNA using primers optimized for insectivore diets, and used Illumina sequencing to characterize the taxonomic composition and diversity of prey. We found 50 unique prey taxa in lady beetle guts from eight arthropod orders (mostly flies, true bugs, and thrips). Lady beetles in landscapes with a greater proportion of crops were slightly more likely to have prey in their gut, and community‐level prey richness was strongly positively correlated with surrounding landcover diversity. This effect was dampened slightly in high‐crop landscapes, likely due to a smaller prey species pool available to predators. Our results enhance knowledge of lady beetle trophic ecology and demonstrate that supplementation of diets through increased habitat diversity may be an important mechanism for the success of mobile generalists in complex landscapes.

## INTRODUCTION

Increasing the complexity of farmed landscapes has the potential to conserve biodiversity and enhance ecosystem services (Haan et al., 2021; Sirami et al., 2019; Tscharntke et al., 2005). Landscape supplementation and complementation are two proposed mechanisms by which complex landscapes may be more favorable to mobile fauna that can track spatially variable resources (Dunning et al., 1992). Landscape supplementation is the process in which an organism benefits from accessing substitutable resources (e.g., different types of food) in adjacent habitat patches in a mixed landscape. For example, wild bees may forage for nectar and pollen in meadows as well as adjacent flowering crop fields (Mallinger et al., 2016; Mandelik et al., 2012; Martins et al., 2018). Landscape complementation occurs when an organism accesses non‐substitutable resources (e.g., food and shelter) in distinct habitat types in a landscape (Clake et al., 2022; Haynes et al., 2007; Ouin et al., 2004).

Landscape elements with high potential to contribute to resource complementation and supplementation include non‐crop habitat and a diversity of landcover types. In landscapes with less cropland and more habitat diversity, mobile organisms may be more likely to have access to multiple limiting resources in close proximity, contributing to enhanced fitness and population stability (Dunning et al., 1992). If organisms benefiting from these processes also provide ecosystem services (e.g., pollination, predation of crop pests) landscape complexity can also enhance crop production and decrease reliance on agricultural inputs that substitute the functions of beneficial organisms (e.g., managed honey bees, chemical pesticides; Holzschuh et al., 2012; Mansier & van Rijn, 2024; Meehan et al., 2011).

The realization of such benefits depends upon the life histories of the particular organisms involved and the dimension of landscape complexity under consideration (Haan et al., 2021). For example, species that have wider diet breadths may be more likely to benefit from retention of uncropped areas or increasing crop diversity, since they can take advantage of trophic resources in multiple crop and non‐crop habitat types (Iuliano & Gratton, 2020). Evaluating the extent to which particular species actually use resources from distinct habitats, and if so which ones, could provide valuable knowledge for more effective agroecological landscape design.

Lady beetles (family Coccinellidae) are common insect predators in agricultural systems that often contribute to natural pest control (Costamagna & Landis, 2006; Obrycki et al., 2009; Obrycki & Kring, 1998) (but see Kindlmann et al., 2015). While often thought of as specialists on small, soft‐bodied insects (e.g., aphid or scales), lady beetles are known to opportunistically consume a variety of alternative prey (Evans, 2009; Romero et al., 2023; Weber & Lundgren, 2009). Lady beetles can be reliable predators of target prey in controlled environments, but realized foraging in real landscapes is harder to predict and measure. Controlled laboratory experiments have demonstrated that dietary diversity can mitigate negative effects of low‐quality prey on lady beetles, in some cases shortening development time, improving growth, and/or increasing fecundity (Evans et al., 1999; Harwood & Obrycki, 2005; Stowe et al., 2021). Thus, landscapes that provide lady beetles with a diverse buffet should also enhance their populations and predation services (Harwood & Obrycki, 2005; Iuliano & Gratton, 2020; Wyckhuys et al., 2024) (but see Koss & Snyder, 2005). Simplified landscapes have been shown to have negative effects on lady beetle body condition (Tiede et al., 2022) and alter their gut microbiome, likely mediated by food availability and diversity (Tiede et al., 2017). Yet, to our knowledge, no study has yet linked landscape conditions to lady beetle diet composition.

Real‐world arthropod diets and predator–prey interaction networks are notoriously difficult to characterize. Traditional methods including direct observation (Groden et al., 1990; Warreen & Tadic, 1967), gut dissection and microscopy (Triltsch, 1997, 1999), frass analysis (Davidson & Evans, 2010), and stable isotope analysis (Forbes & Gratton, 2011) are time consuming, prone to inaccuracies, and biased against soft‐bodied prey items and early insect life stages (eggs and larvae) that may comprise large portions of arthropod predator diets. Advances in next‐generation sequencing (NGS) and DNA metabarcoding techniques (Alberdi et al., 2019; Traugott et al., 2013) have enabled ecologists to characterize animal diets and assess biological control services provided by predatory arthropods (Furlong, 2015; González‐Chang et al., 2016; Lue et al., 2022). Compared to traditional approaches, metabarcoding requires lower observer effort per sample once laboratory and bioinformatic pipelines are established, accommodates hundreds of individuals simultaneously at relatively modest cost per specimen, and generally recovers a broader range of prey with higher taxonomic resolution, especially for soft‐bodied and cryptic species (King et al., 2008; Pompanon et al., 2012). The method may still be limited by incomplete reference databases, primer biases, and challenges translating sequence reads into quantitative predation estimates (Clare, 2014). Several studies have employed these tools to analyze lady beetle diets (Ammann et al., 2020; Kim et al., 2022; Romero et al., 2023; Yadav & Pervez, 2025), yet limited spatial replication and geographic extent have constrained their ability to make inferences about how diets vary with local habitat or landscape context.

In this study, we used NGS of lady beetles collected from agricultural landscapes in southern Wisconsin to explore sources of variation in the composition and richness of their diets. Specifically, we investigated how diets varied by lady beetle species and local and landscape habitat context. In two growing seasons, we collected lady beetles of eight species from six habitat types in landscapes selected to span gradients of proportion cropland and land cover diversity in the surrounding area. We predicted that prey detection rates and dietary richness would be higher in non‐crop (grassland and woodland) sites, negatively correlated with the proportion of cropland in the landscape, and positively correlated with landscape diversity.

## METHODS

### Field sampling

We collected adult lady beetles in 2 years (2019 and 2021) from agricultural fields and natural habitat areas in Southern Wisconsin, USA. There are at least 15 native species of lady beetles (Coccinellidae) in the region (Gardiner et al., 2021; Gardiner, Landis, Gratton, Schmidt, et al., 2009; Iuliano et al., 2024), but a large portion of the community comprises introduced species. We restricted our collection to the most commonly encountered species: the native *Coleomegilla maculata*, *Cycloneda munda*, *Hippodamia convergens*, and *Hippodamia parenthesis*, and the introduced *Harmonia axyridis*, *Coccinella septempunctata*, *Propylea quatuordecimpunctata*, and *Hippodamia variegata*. While each of these species has unique life history traits, they all display some level of polyphagy (i.e., consume prey from multiple families) and were encountered in several habitat types during field surveys. We collected only adult beetles, which we assumed would be able forage across multiple habitat patches in the landscape (unlike the less‐mobile larvae).

We sampled in six dominant land cover classes found in our study region, grouped into crop and non‐crop habitats. Crops included (1) corn (*Zea mays*); (2) soybean (*Glycine max*); (3) alfalfa (*Medicago sativa*); (4) “small grains” including wheat (*Triticum* spp.), oats (*Avena sativa*), and rye (*Secale cereale*). Non‐crop habitats included perennial pastures, herbaceous wetlands, and prairies—classified together as (5) “grasslands” and (6) “woodlands” consisting of woodlots and forests. These habitats represented >90% of the total area across our replicate landscapes. Collection sites were designated in 17 landscapes in each of the two study years (see Iuliano et al., 2024 for a more detailed description of survey design). Adult lady beetles were sampled opportunistically using sweep nets and aspirators (BioQuip, Rancho Dominguez, CA) across 5–7 site visits between May and September each year. We attempted to collect at least 2 and up to 10 beetles per species per sampling event. Upon collection, lady beetles were transferred to individual microcentrifuge tubes and placed on ice. Samples were brought back to the lab and stored at −20°C until further processing.

### Sample processing and DNA sequencing

We extracted DNA from 509 whole lady beetles using the DNeasy Blood & Tissue Kits (Qiagen, Valencia CA, USA). Extracted DNA was amplified using primers specific to the mitochondrial cytochrome c‐oxidase subunit I region (COI) designed for analysis of insectivorous animal diets (ANML primer pair, LCO1490 and CO1‐CFMRa; Jusino et al., 2019). Amplification conditions were as follows: 95°C for 15 min, 35 cycles of 94°C for 30 s, 51°C for 30 s and 72°C for 90 s, and a final extension of 72°C for 10 min. Random samples of PCR products were visualized via gel electrophoresis to confirm amplification. All samples were then plated, with 2–6 negative controls (blanks) included on each 96‐well plate.

PCR products were submitted to the University of Wisconsin Biotechnology Center for library preparation and sequencing. Samples were sequenced in four pooled runs using Illumina NovaSeq 6000 with paired‐end, 150 base pair sequencing. Targeted read depth was 3 million reads per 96‐well plate (approximately 30,000 reads per sample).

### Bioinformatics and data filtering

The data generated from the Illumina sequencing were processed and analyzed using Quantitative Insights Into Microbial Ecology (QIIME2) version 22020.8 (Bolyen et al., 2019). Sequencing reads were denoised and quality filtered using the denoising program Divisive Amplicon Denoising Algorithm (DADA2; Callahan et al., 2016). DADA2 was used to trim low‐quality bases, filter out noisy sequences, correct errors in marginal sequences, merge overlapping paired‐end reads, and remove chimeric sequences and singletons and then dereplicate those sequences. The resultant dereplicated sequences are termed as “Amplicon sequence variant (ASV)”. Sequence variants were aligned and masked using MAFFT (Katoh et al., 2002), and the phylogenetic tree of the ASV's created using FastTree (Price et al., 2009). Low‐frequency reads and index bleed through between sequence runs estimated to be 0.1% of the reads were removed from the analysis. Finally, taxonomy was assigned using a hybrid method, a combination of VSEARCH (Rognes et al., 2016), UTAX, and SINTAX (Edgar, 2016) from the Amplicon Toolkit Pipeline (AMPtk; Palmer et al., 2018) to the mitochondrial COI region. The COI database used for the taxonomy classification consists of both arthropod and chordate and is pulled from the BOLDv4 database (Ratasingham & Hebert, 2007). All non‐arthropod reads were excluded from the analysis.

Following initial sequence processing and quality filtering, Actual read depth per sample ranged from 118 to 629,917 reads, with an average of 271,614, 10,652, 41,759, and 14,944 reads per sample for each of the four sequencing runs. Eight samples had fewer than 500 reads and were removed from the analysis.

After reads were assigned to operation taxonomic units (OTUs) separately for each pool, reads with matching OTUs were aggregated and data from all four pools were combined for subsequent quality filtering. We constructed a prey OTU presence/absence matrix following best practices for controlling false positives in metabarcoding data (Deagle et al., 2019; Drake et al., 2022). We filtered reads by removing those less than or equal to the maximum read count in negative controls per OTU per sequencing run and used a conservative sample‐based threshold of 1% of total reads per OTU per sample (Drake et al., 2022). Due to high levels of nonhost lady beetle reads (particularly *H. axyridis*) in samples and negative controls (likely due to contamination), we excluded all lady beetle reads from our analysis, and thus, we were not able to evaluate the potential for intraguild predation. We also removed reads of lady beetle parasitoids (e.g., *Dinocampus coccinellae*) and taxa which are known not to occur in the study region.

### Landscape data

Landscape data were derived from the USDA Cropland Data Layer (CDL) available for each year of the study (USDA NASS, n.d.). To reduce the influence of spurious classification and functionally synonymous classes on calculated landscape metrics, we reclassified landcover maps to the seven most common classes in the region (corn, soybean, alfalfa, small grains, other crops, grassland/pasture, woodland). We used the landscapemetrics package (Hesselbarth et al., 2019) to calculate the proportion of cropland (sum of crop cover classes, excluding grassland/pasture and woodland) and landscape diversity (based on all seven cover classes) in a 250‐m buffer around sampling locations using Simpson's diversity index (following Gardiner, Landis, Gratton, DiFonzo, et al., 2009; Tiede et al., 2022). The Simpson index is less sensitive to rare classes than habitat richness or Shannon diversity, making it a more conservative estimate of landscape diversity. We selected 250‐m buffers as a reasonable maximum spatial extent across which we might expect foraging lady beetle adults to travel over the detectability half‐life of prey DNA in their gut (typically <12 h; Chen et al., 2000; Greenstone et al., 2014).

### Data analysis

All data handling and statistical analyses were conducted in R version 4.3.1 (R Core Team, 2023) with RStudio version 2023.06.0+421 (RStudio Team, 2023). Model assumptions were validated using the DHARMa package (Hartig & Lohse, 2022). Data were visualized using the packages ggplot2 (Wickham, 2016), ggeffects (Lüdecke et al., 2023), and bipartite (Dormann et al., 2022).

Due to low sample abundance and low prey detection rates for lady beetle species other than *C. maculata* (Table 1), we only present descriptive illustrations rather than formal statistical tests of prey diets among lady beetle species. To evaluate the effects of local habitat type and landscape context on lady beetle diets, we aggregated all beetles of each species collected per site‐year to calculate population‐wide prey detection rates (proportion of beetles with at least one prey item detected) and prey taxa richness for beetles where prey were present (i.e., prey richness >0), which were used as response variables in generalized linear mixed models constructed using the glmmTMB package (Brooks et al., 2017). We used a model selection approach to evaluate the importance of local landcover (crop vs. non‐crop habitat) and landscape context (% cropland and landcover diversity in a 250 m radius) for lady beetle diets. Full models contained all three standardized predictors and their interactions as fixed effects. Collinearity checks showed minimal correlation between predictors (all variance inflation factors <5; Appendix S1: Table S1, Figure S1). We included lady beetle species and location nested within year (to account for nonindependence) as random intercepts and sample size (number of beetles per species collected at a location over the year) as weights. We modeled prey presence with a binomial distribution, and prey richness with a Poisson distribution. We used the dredge function in the package MuMIn (Bartoń, 2023) to determine which combination of predictors best explained variation in lady beetle diet data using corrected Akaike's information criterion (AICc). We report statistics for all models and use the top model to visualize results.

**TABLE 1:** Lady beetle species included in the study by origin, sample size, prey detection rate (≥1 prey), and number of unique OTUs identified over the 2 years of the study.

Species	Origin	*n*	Detection rate (%)	Prey OTUs
*Coleomegilla maculata*, DeGeer	Native	281	21	47
*Harmonia axyridis*, Pallas	Introduced	81	5	7
*Coccinella septempunctata*, L.	Introduced	45	18	5
*Propylea quatuordecimpunctata*, L.	Introduced	29	17	3
*Hippodamia variegata*, Goeze	Introduced	28	4	1
*Cycloneda munda*, Say	Native	13	15	1
*Hippodamia convergens*, Guérin‐Méneville	Native	9	11	1
*Hippodamia parenthesis*, Say	Native	6	17	1
Overall		492	17%	50

## RESULTS

### Lady beetle and prey species

After sample quality filtering, we retained 492 lady beetles from eight species for analysis (Table 1). Samples were dominated by *C. maculata* (57%), which was also a dominant species in standardized abundance surveys (Iuliano et al., 2024), followed by *H. axyridis* (16%), *C. septempunctata* (9%), *P. quatuordecimpunctata* (6%), and *H. variegata* (6%); other species combined comprised the final 6% of samples. Average prey detection rate across all species was 17%, with the highest detection in *C. maculata* (21%) and the lowest in *H. variegata* (4%).

Lady beetles were collected in 159 distinct site‐year combinations, 59 of which included at least one lady beetle with at least one prey species detected. Most lady beetles came from corn fields (*n* = 52 site‐years), followed by grasslands (*n* = 28), alfalfa fields (*n* = 26), soybean fields (*n* = 23), woodlands (*n* = 17), and small grain fields (*n* = 13). The sample size of lady beetles per site‐year ranged from 1 to 22 individuals.

**FIGURE 1:**
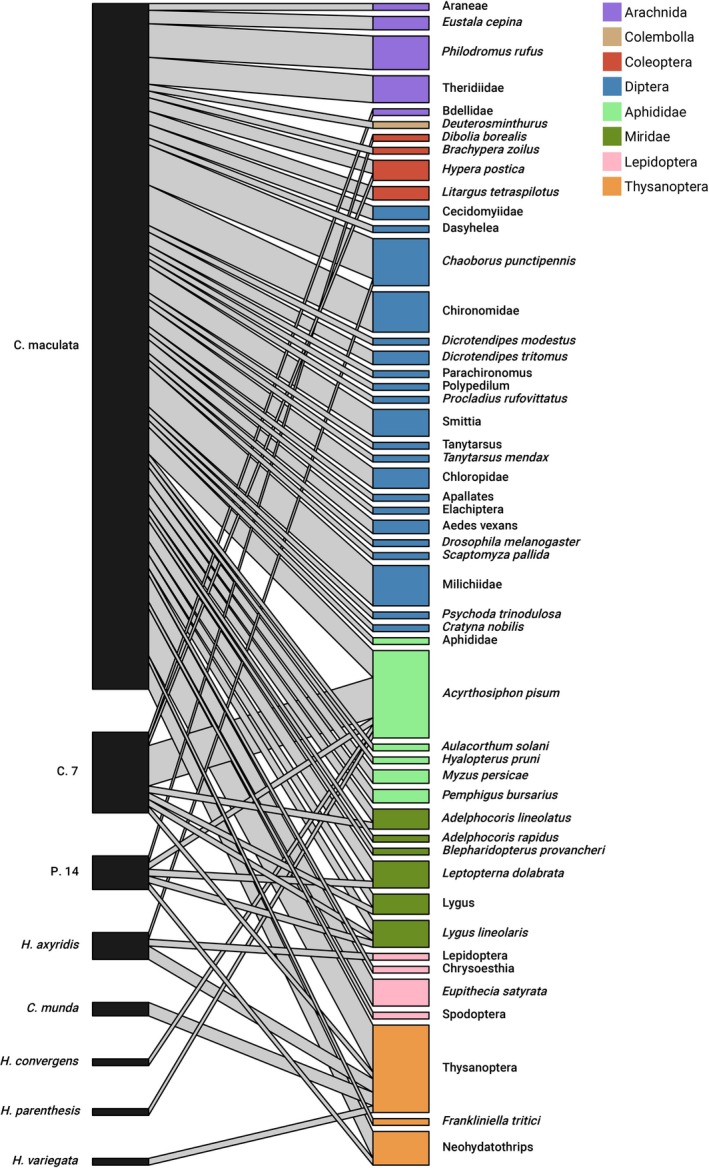
Bipartite network diagram of lady beetle prey trophic relationships from DNA sequencing data. Colors represent arthropod orders of prey taxa, with the exception of hemipterans which are divided into aphids (light green) and mirid bugs (dark green). Labels correspond to the lowest taxonomic level identified. C.7 is *C. septempunctata*; P.14 is *P. quatuordecimpunctata*.

We detected 50 unique prey OTUs from eight arthropod orders in our samples (Figure 1). Prey taxa were dominated by flies (35% of detected prey), true bugs (aphids and mirid bugs, 28%), and thrips (15%).

### Local habitat and landscape context

**FIGURE 2:**
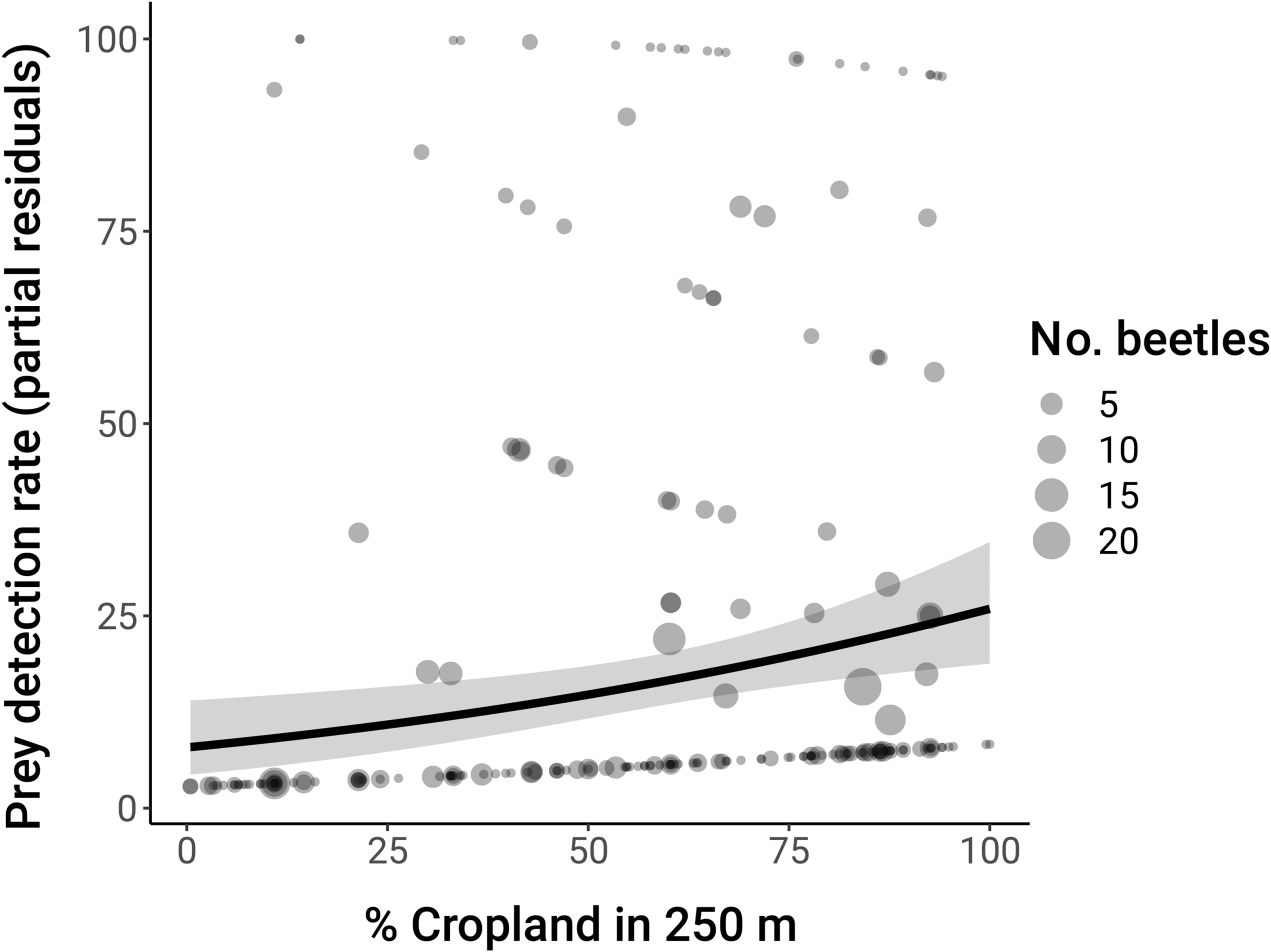
Predicted effects of proportion cropland within 250 m of collection sites on prey detection rates in the sampled lady beetle community. Points are partial residuals averaging over the random effects of species and site nested within year. Point sizes represent the number of lady beetles sequenced per site. Shaded area depicts 95% CI.

The top model for prey detection at the site level (proportion of beetles with prey detected) included only the percent of cropland within 250 m of the sampling site (Table 2, Figure 2). Prey detection increased modestly with increasing proportion cropland (% cropland effect, β = 0.37, CI: 0.09, 0.65). Models with ΔAICc <2 also included landscape diversity and its interaction with percent cropland (Table 2), but 95% CIs for these terms contained zero, suggesting they were not important predictors of prey detection.

**TABLE 2:** Comparison of top five candidate models for prey detection in lady beetle samples.

Model rank	Habitat (natural vs. crop)	Percent cropland	Landscape diversity	Percent cropland × habitat	Landscape diversity × habitat	Percent cropland × landscape diversity	Percent cropland × landscape diversity × habitat	df	ΔAICc
1		**0.37 (0.09, 0.65)**						**5**	**0.00**
2		0.35 (0.06, 0.65)	0.32 (−0.08, 0.71)			−0.34 (−0.70, 0.03)		7	0.39
3		0.38 (0.10, 0.67)	0.07 (−0.19, 0.35)					6	1.79
4	0.07	0.38						6	2.06
5	0.08	0.36	0.32			−0.34		8	2.46

*Note*: All models included an intercept and random intercepts for location nested within year and lady beetle species. Predictors included local habitat type, percent cropland, and landscape diversity within 250 m, and their interactions. Values represent standardized coefficients for continuous variables. Total degrees of freedom and difference in Akaike's information criterion corrected for small sample sizes (ΔAICc) from the top model (in bold) are also shown. 95% CIs are shown for models with ΔAICc <2.

**FIGURE 3:**
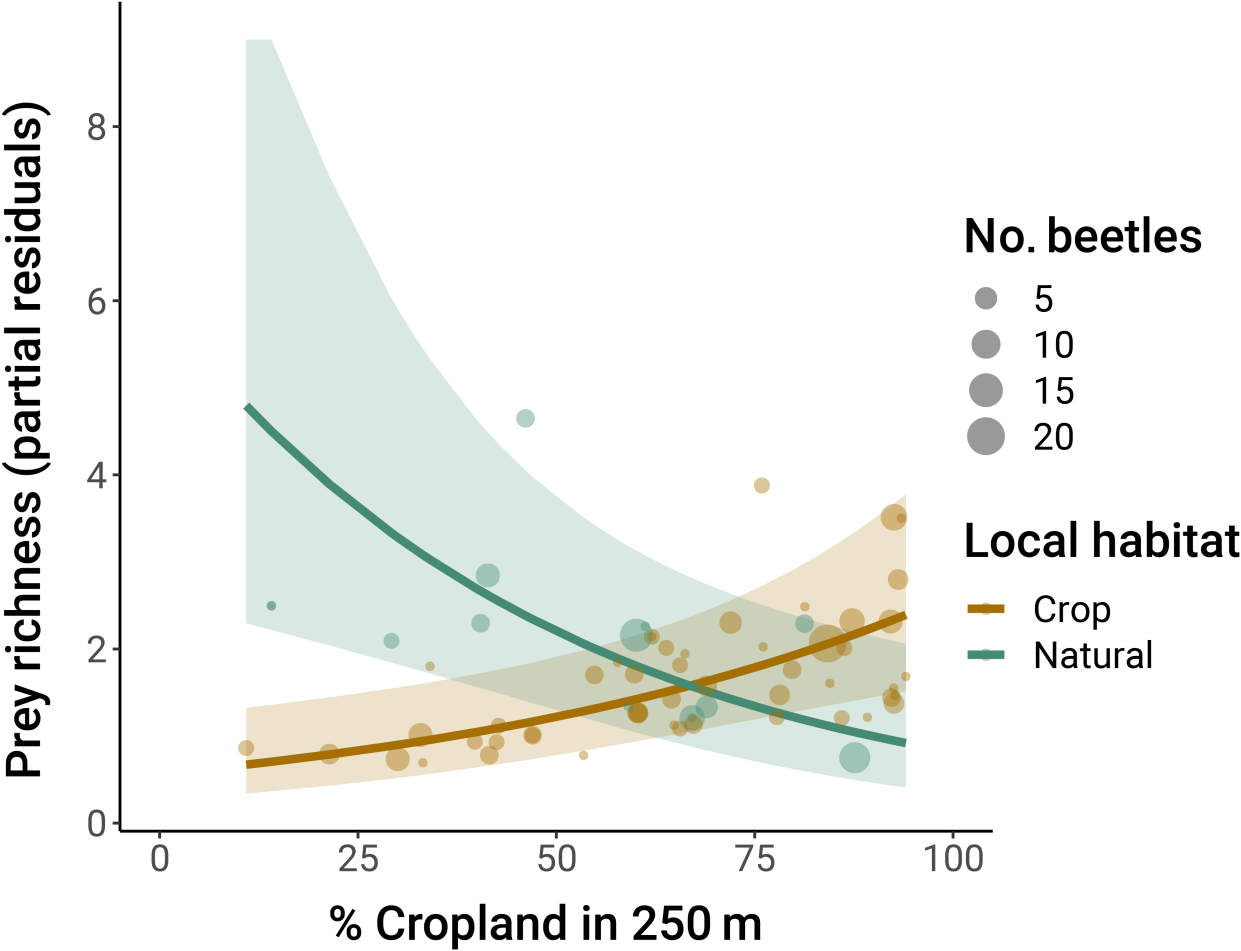
Predicted effects of the proportion cropland within 250 m of collection sites on prey taxa richness in the sampled lady beetle community, by local habitat type. Points are partial residuals holding landscape diversity at its mean and averaging over the random effects of species and site nested within year. Point sizes represent the number of lady beetles sequenced per site. Colors represent local habitat category. Shaded areas depict 95% CIs.

The top model for prey taxa richness included habitat type, proportion cropland within 250 m, and their interaction, as well as landscape diversity and its interaction with proportion cropland (Table 3). Increasing proportion cropland in the surrounding landscape was positively correlated with prey richness in lady beetles collected from crop habitats (β = 0.34, CI: 0.14, 0.54) but negatively correlated with prey richness for beetles collected from natural areas (β = −0.44, CI: −0.80, −0.09; Figure 3).

**TABLE 3:** Comparison of top five candidate models for prey richness in lady beetle samples.

Model rank	Habitat (natural vs. crop)	Percent cropland	Landscape diversity	Percent cropland × habitat	Landscape diversity × habitat	Percent cropland × landscape diversity	Percent cropland × landscape diversity × habitat	df	ΔAICc
1	**0.13 (−0.33, 0.58)**	**0.34 (0.14, 0.54)**	**0.60 (0.35, 0.85)**	**−0.78 (−1.14, −0.43)**		**−0.32 (−0.54, −0.10)**		**9**	**0.00**
2	0.26	0.39	0.71	−0.81	−0.19	−0.40		10	2.12
3	0.28	0.37	0.66	−0.77	−0.13	−0.35	−0.38	11	4.28
4	0.02	0.21	0.31	−0.63				8	5.08
5	−0.10	0.19	0.27	−0.65	0.22			9	6.15

*Note*: All models included an intercept and random intercepts for location nested within year and lady beetle species. Predictors included local habitat type, percent cropland, and landscape diversity within 250 m, and their interactions. Values represent standardized coefficients. Total degrees of freedom and difference in Akaike's information criterion corrected for small sample sizes (ΔAICc) from the top model (in bold) are also shown. 95% CIs are shown for models with ΔAICc <2.

**FIGURE 4:**
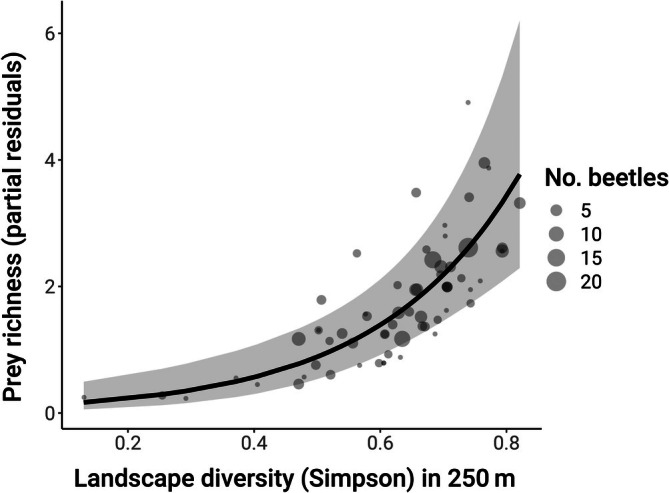
Predicted effects of landscape diversity within 250 m of collection sites on prey taxa richness in the sampled lady beetle community. Points are partial residuals, holding proportion cropland at its mean and averaging over local habitat category and the random effects of species site nested within year. Point sizes represent the number of lady beetles sequenced per site.

**FIGURE 5:**
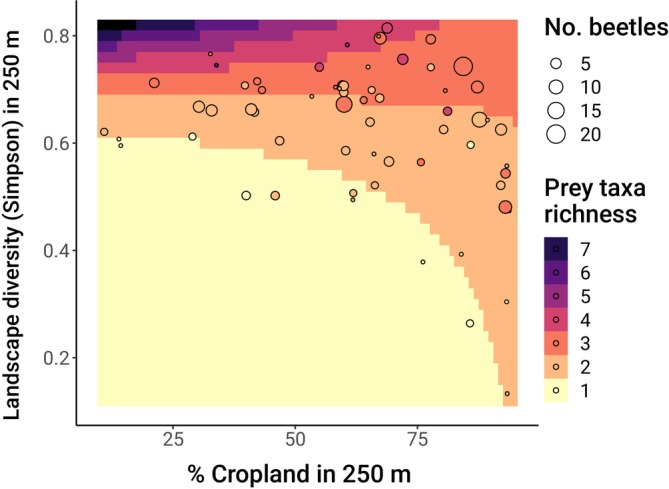
Predicted interaction of proportion cropland and landscape diversity within 250 m of collection sites on prey taxa richness in the sampled lady beetle community. Background colors represent model‐predicted prey richness. Points represent partial residuals of observed prey richness, averaging over local habitat type and the random effects of species site nested within year. Point sizes represent the number of lady beetles sequenced per site.

In addition, the strongest effect was the positive relationship between landscape diversity and prey taxa richness (landscape diversity effect, β = 0.60, CI: 0.35, 0.85; Figure 4). This effect was further mediated by proportion cropland: At high levels of cropland in the surrounding area, the positive effect of landscape diversity on prey richness slightly decreased (% cropland × landscape diversity effect, β = −0.32, CI: −0.54, −0.10; Figure 5).

## DISCUSSION

The ability of mobile consumers to take advantage of resources from multiple habitats is a key assumption underlying calls to increase landscape complexity for the promotion of biodiversity‐based ecosystem services (Jaworski et al., 2023; Mansier & van Rijn, 2024; Schellhorn et al., 2015; Tscharntke et al., 2021, 2025; Wyckhuys et al., 2025). Validating this assumption requires characterizing real‐world diets of service‐providing species and their response to habitat conditions and landscape structure. In this study, we sought to investigate how prey taxa consumed by predatory lady beetles varied by beetle species, local foraging habitat, and landscape complexity. We found that lady beetles collected from more diverse, cropped landscapes were more likely to forage on a broader array of prey taxa. To our knowledge, this study is the first to document a correlation between the diversity of prey consumed by predatory insects and the landcover diversity of the surrounding area, a finding consistent with the idea that landscape supplementation can be an important ecological process for populations of service‐providing organisms (Dunning et al., 1992). Our results contribute to the growing body of evidence demonstrating that landscape context is an important driver of trophic interactions for mobile consumers (Donkersley et al., 2014; Penn et al., 2017; Saqib et al., 2022) and suggests a mechanism by which complex landscapes contribute to the conservation of predatory insects and their associated pest control services.

### Evidence of landscape complementation in lady beetle diets

Prey detection and taxa richness in the diet of the mobile predators we examined were predicted by the interaction of several local and landscape habitat variables. The proportion of the surrounding landscape covered by cropland was the only variable that had a measurable effect on prey detection rates, with beetles from high‐crop landscapes slightly more likely to contain prey in their guts at the time of collection. This may suggest that lady beetles forage more actively in these landscapes, reflecting high densities of preferred prey in crop fields (Donaldson et al., 2007; Yasuda & Ishikawa, 1999), or that simpler vegetation architecture in cropped landscapes enables lady beetles to spend more time eating and less time navigating and searching for prey (Pervez & Yadav, 2018). In either case, cropped habitats appear to be an important source of prey for lady beetles in these landscapes.

Prey richness in the aggregate sample of lady beetles at a given site was positively correlated with the diversity of land covers surrounding that site, regardless of the local habitat type (crop or natural) in which the beetles were collected. This finding provides indirect support for inter‐habitat movement of lady beetles and is consistent with the hypothesis that generalist predators are able to take advantage of the eclectic buffet that complex landscapes offer (i.e., landscape complementation; Dunning et al., 1992; Iuliano & Gratton, 2020). It also may partially explain why lady beetle abundance is often correlated with metrics of landscape complexity, at least for some species (Croy et al., 2023; Gardiner, Landis, Gratton, DiFonzo, et al., 2009; Gardiner, Landis, Gratton, Schmidt, et al., 2009; Iuliano et al., 2024).

Because most of the prey taxa we detected in lady beetle guts were not habitat specialists (i.e., they can be found in multiple land cover types), we were unable to make statistical inferences about allochthonous prey (i.e., prey from habitats other than that in which a given beetle was collected). However, in our data, there are several examples of lady beetles collected from one habitat that contained DNA from prey only present in other habitats. For example, from soybean fields, we collected two *C. septempunctata* individuals whose guts contained *Acyrthosiphon pisum* (pea aphid), which in this region is primarily associated with alfalfa fields; in a corn field, one *C. maculata* individual contained *Hypera postica* (alfalfa weevil) which is a specialist on alfalfa; and in alfalfa, one *C. maculata* and one *P. quatuordecimpunctata* that contained *Leptopterna dolabrata* (meadow plant bug), a grass specialist. These records provide evidence of lady beetle foraging across the landscape. An alternative (if less likely) explanation could be that the lady beetles encountered prey in the “wrong” habitat as the prey were traversing the landscape. More intensive sampling of fewer sites could offer a clearer picture of cross‐habitat foraging. There are, however, inherent constraints to metabarcoding approaches for detecting spatially complementary resource use given that the half‐life of prey DNA in predator guts may be shorter than the timespan within which predators typically move to a new habitat patch.

### Interactions among local and landscape habitat conditions

For lady beetle communities collected from crop fields, a greater proportion of cropland in the surrounding landscape was positively associated with prey taxa richness, while for communities collected from grasslands and woodlands, the opposite was true. This may reflect context‐specific habitat use patterns by lady beetles, whereby beetles in crop‐dominated landscapes prefer to forage on the abundant prey in crop fields and use seminatural areas as refuge from disturbance (Honěk, 2012; Iuliano et al., 2024), while lady beetles in low‐crop landscapes rely more heavily on seminatural areas for food. Furthermore, the beetles we collected for this study came from ground cover and understory vegetation, missing lady beetles in the canopy where species composition and behavior might be different (Cottrell, 2017; Honěk, 2012; Ulyshen & Hanula, 2007).

More cropland in the landscape also dampened the positive effect of landscape diversity on prey taxa richness. This likely reflects an inherent ecological difference between high‐habitat diversity/low‐crop landscapes and high‐habitat diversity/high‐crop landscapes. Low‐crop landscapes in our study were dominated by seminatural grasslands and woodland patches, which contain more diverse plant and corresponding arthropod communities than crop fields (Robertson et al., 2012; Werling et al., 2014). Accordingly, beetles foraging in these landscapes likely had access to a greater breadth of prey taxa than beetles in similarly diverse, crop‐dominated landscapes. In diverse but crop‐dominated landscapes, however, the number of prey species available to foraging lady beetles may be constrained by relatively low arthropod diversity in crop habitats, even when multiple crop types are present.

### Taxonomic diversity of lady beetle diets in agricultural landscapes

Despite relatively low prey detection rates (4%–21% of beetles, depending on species), DNA metabarcoding revealed a remarkably wide diet breadth of the lady beetles in our study, consistent with other research on the trophic interactions of coccinellids (Evans, 2009; Hodek & Evans, 2012; Kim et al., 2022; Romero et al., 2023).

The OTUs most commonly detected in lady beetles included species in the order Diptera (43% of hits), which also had the highest number of unique prey OTUs (52% of OTUs). Past research has drawn attention to the potential importance of flies in lady beetle diets. Using an antibody‐based detection system, Moser et al. (2011) showed that late‐instar larvae of three lady beetle species, including *C. maculata*, can prey upon flies, though detection rates in larvae were much lower (generally <5%) than in this study of adult lady beetles. Kim et al. (2022) and Romero et al. (2023) also found that flies were prey for field‐collected adult lady beetles using DNA metabarcoding, but detection rates were still <13%. Laboratory studies have demonstrated that fruit fly (*Drosophila melanogaster*) larvae (Schultz et al., 2019) and house fly (*Musca domestica*) eggs (Riddick et al., 2014) can serve as high‐quality prey items for commercial rearing of *C. maculata* throughout their entire lifecycle, suggesting that foraging on flies by natural populations is plausible even if difficult to observe.

Several of the fly taxa detected in our samples (e.g., those in the families Chaoboridae and Chironomidae) are aquatic species that reside in freshwater lakes or streams as eggs and larvae. Because these life stages would be inaccessible to lady beetles, it implies that beetles consume adults of these taxa on vegetation, perhaps as they nectar at flowers, get caught on plant trichomes, or if they are sedentary at times of the day when beetles are relatively more active (i.e., due to differences in body size). It is possible that detection of aquatic species in lady beetle diets was due to sample contamination missed by quality filtering. In this analysis, we used a relatively stringent filtering criterion (<1% of OTUs per sample; Drake et al., 2022) for excluding potentially erroneous OTUs. Using an even more extreme cutoff (2%) yielded qualitative similar results in terms of the patterns with local and landscape factors and did not diminish the importance of aquatic Diptera in the diet of these lady beetles. This result underscores the value of conceptualizing agricultural landscapes as an interacting network of terrestrial and aquatic ecosystems linked by the movement of mobile organisms (such as predators and/or prey), and how they could be comanaged to promote insect conservation and the ecosystem services they may provide (Bergerot et al., 2025; Gratton & Vander Zanden, 2009).

Other taxa comprising a large portion of lady beetle diets were hemipterans (aphids and mirid bugs) and thrips, which are well documented as common lady beetle prey. Sixteen prey taxa (32% of OTUs) detected in lady beetle samples were pests known to cause economic damage, including alfalfa weevils (*Hypera postica*), pea aphid (*Acyrthosiphon pisum*), green peach aphid (*Myzus persicae*), tarnished plant bug (*Lygus lineolaris*), and eastern flower thrips (*Frankliniella tritici*). The wide beetle diet breadths found here suggest that lady beetles both contribute to suppression of pests beyond aphids and rely on a multitude of non‐pest species from both crop fields and seminatural habitats to maintain their populations.

The majority of beetles in our study were *Coleomegilla maculata*, a native species that has maintained large populations in the region while other native species have declined due to land use changes and competition from introduced species (Bahlai et al., 2015; Gardiner et al., 2021; Lamb et al., 2019; Perry et al., 2024). Our findings support speculation that *C. maculata*'s relative stability in the face of invasion could be due to its ability to exploit a wide variety of prey, thus alleviating competitive pressures.

Because our samples were dominated by this species, we are unable to make reliable comparisons of lady beetle diets by species. For example, we only detected a single prey taxon in a single lady beetle for *H. variegata* (thrips), *H. convergens*, and *H. parenthesis* (*A. pisum*, pea aphid). Accordingly, we caution that conservative interpretation of our results—as primarily a reflection of the dietary patterns in *C. maculata*—is warranted. More targeted sampling of rarer lady beetle species is required to accurately characterize and contrast their diets. Further refinement of laboratory techniques may also be helpful, given the surprisingly low prey detection rate (5%) for the dominant generalist *H. axyridis*. High DNA read counts of this lady beetle species itself (excluded from analysis here) may indicate that it amplified more readily than DNA from co‐occurring taxa, potentially obscuring prey detection in samples of *H. axyridis*. *H. axyridis* contamination also prevented us from accurately assessing cannibalism and intraguild predation, which can be high among coccinellids (Yadav & Pervez, 2025). Moreover, differences in feeding or digestion rates among species could favor species with more frequent feeding or slower gut‐passage times of prey (Greenstone et al., 2014; Uiterwaal & DeLong, 2020).

## CONCLUSION

The findings of this study contribute to knowledge of lady beetle trophic ecology and the mechanisms by which landscape structure influences populations of mobile consumers. The strong correlation observed here between landcover diversity and dietary richness indicates that landscape complementation can help buffer against temporal or spatial variability of prey for some lady beetle species, and potentially other generalist predators. By identifying the interacting contributions of natural history, local habitat, and landscape context to patterns of resource use by beneficial species, we may refine agricultural landscape design to sustain mobile consumers and their associated ecosystem services. Future metabarcoding studies designed to compare the relative diet breadths of different species could further clarify contrasting population trajectories of lady beetles in response to biological invasions and landscape change. Furthermore, multivariate analysis techniques could be applied to similar dietary datasets to test how prey community composition changes across landscape gradients. Such research will deepen our understanding of the roles of landscape composition and diversity that underlie the maintenance of biological pest control and other biodiversity‐based services in agricultural landscapes.

## AUTHOR CONTRIBUTIONS

Benjamin Iuliano and Claudio Gratton conceived of and designed the study. Benjamin Iuliano led field work, sample processing, data analysis, and wrote the first manuscript draft. Claudio Gratton reviewed and contributed to subsequent drafts.

## CONFLICT OF INTEREST STATEMENT

The authors declare no conflicts of interest.

## Supporting information


Appendix S1.

## Data Availability

Data and code (Iuliano & Gratton, 2026) are available in Dryad at https://doi.org/10.5061/dryad.wstqjq31m.
