# Supplementary material for: Complex landscapes, complex diets: DNA metabarcoding reveals lady beetle prey richness increases with landcover diversity
Source: Ecol Appl. 2026 May 11;36:e70248. doi: 10.1002/eap.70248 (PMC13158908; doi:10.1002/eap.70248)
Supplement: Supplementary file 1 — Appendix S1. [file EAP-36-e70248-s001.pdf]

Appendix S1

Complex landscapes, complex diets: DNA metabarcoding reveals lady beetle prey richness increases with landcover diversity

Benjamin Iuliano, Claudio Gratton

Ecology

Figure S1. Illustration of predictor variables in models of local and landscape effects on lady beetle diets. Each point represents a site-year.

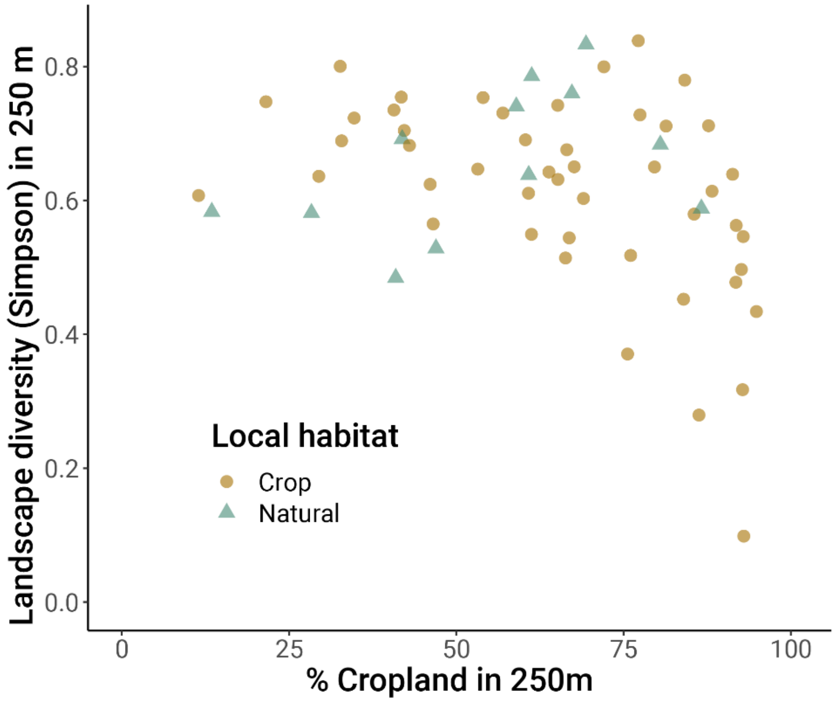

Table S1. Variance Inflation Factors (VIFs) for predictor variables in models of local and landscape effects on lady beetle diets.

	**VIF**
Habitat	2.86
% Cropland	1.99
Landscape diversity	3.99
Percent Cropland × Habitat	2.11
Landscape diversity × Habitat	2.64
Percent Cropland × Landscape diversity	3.03
Percent Cropland × Landscape diversity × Habitat	1.92
